# Ex vivo cultivated retinal pigment epithelial cell transplantation for the treatment of rabbit corneal endothelial dysfunction

**DOI:** 10.1186/s40662-023-00351-4

**Published:** 2023-08-02

**Authors:** Chunxiao Dong, Dulei Zou, Haoyun Duan, Xiangyue Hu, Qingjun Zhou, Weiyun Shi, Zongyi Li

**Affiliations:** 1 Department of Medicine, Qingdao University, Qingdao, 266071 China; 2 Eye Institute of Shandong First Medical University, State Key Laboratory Cultivation Base, Shandong Provincial Key Laboratory of Ophthalmology, Qingdao, 266071 China; 3 Eye Hospital of Shandong First Medical University (Shandong Eye Hospital), Jinan, 250000 China; 4 School of Ophthalmology, Shandong First Medical University, Jinan, 250000 China

**Keywords:** Corneal endothelium, Retinal pigment epithelium, Corneal endothelial dysfunction, Cell transplantation, Stem cell therapy

## Abstract

**Objective:**

Stem cell therapy is a promising strategy for the treatment of corneal endothelial dysfunction, and the need to find functional alternative seed cells of corneal endothelial cells (CECs) is urgent. Here, we determined the feasibility of using the retinal pigment epithelium (RPE) as an equivalent substitute for the treatment of corneal endothelial dysfunction.

**Methods:**

RPE cells and CECs in situ were obtained from healthy New Zealand male rabbits, and the similarities and differences between them were analyzed by electron microscopy, immunofluorescent staining, and quantitative real-time reverse transcription polymerase chain reaction (qRT-PCR). Rabbit primary RPE cells and CECs were isolated and cultivated ex vivo, and Na+/K+-ATPase activity and cellular permeability were detected at passage 2. The injection of cultivated rabbit primary RPE cells, CECs and human embryonic stem cell (hESC)-derived RPE cells was performed on rabbits with corneal endothelial dysfunction. Then, the therapeutic effects were evaluated by corneal transparency, central corneal thickness, enzyme linked immunosorbent assay (ELISA), qRT-PCR and immunofluorescent staining.

**Results:**

The rabbit RPE cells were similar in form to CECs in situ and ex vivo, showing a larger regular hexagonal shape and a lower cell density, with numerous tightly formed cell junctions and hemidesmosomes. Moreover, RPE cells presented a stronger barrier and ionic pumping capacity than CECs. When intracamerally injected into the rabbits, the transplanted primary RPE cells could dissolve corneal edema and decrease corneal thickness, with effects similar to those of CECs. In addition, the transplantation of hESC-derived RPE cells exhibited a similar therapeutic effect and restored corneal transparency and thickness within seven days. qRT-PCR results showed that the expressions of CEC markers, like CD200 and S100A4, increased, and the RPE markers OTX2, BEST1 and MITF significantly decreased in the transplanted RPE cells. Furthermore, we have demonstrated that rabbits transplanted with hESC-derived RPE cells maintained normal corneal thickness and exhibited slight pigmentation in the central cornea one month after surgery. Immunostaining results showed that the HuNu-positive transplanted cells survived and expressed ZO1, ATP1A1 and MITF.

**Conclusion:**

RPE cells and CECs showed high structural and functional similarities in barrier and pump characteristics. Intracameral injection of primary RPE cells and hESC-derived RPE cells can effectively restore rabbit corneal clarity and thickness and maintain normal corneal function. This study is the first to report the effectiveness of RPE cells for corneal endothelial dysfunction, suggesting the feasibility of hESC-derived RPE cells as an equivalent substitute for CECs.

**Supplementary Information:**

The online version contains supplementary material available at 10.1186/s40662-023-00351-4.

## Background

The corneal endothelium is a monolayer of regular hexagonal cells that maintains corneal hydration and transparency through pump and barrier functions [1, 2]. Adult corneal endothelial cells (CECs) exhibit a limited proliferative capacity in vivo. When injured, corneal endothelial defects are compensated for by the migration and enlargement of adjacent cells. Various factors, such as dystrophy, trauma and surgery, can contribute to bullous keratopathy with CECs loss as well as corneal edema and visual impairment [3]. Corneal transplantation is the dominant therapeutic approach for corneal endothelial dysfunction, but transplant-grade donor corneas are severely limited worldwide [4, 5].

Many strategies have been developed for the treatment of corneal endothelial dysfunction, such as drug protective therapy [6–8], artificial endothelial layer [9], corneal endothelial cell-based tissue-engineered construct [10, 11] and corneal endothelial cell-based cell injection [12, 13]. Professor Kinoshita’s team has reported on the clinical trial of intracameral injection of cultivated human CECs with Rho-associated, coiled-coil containing protein kinase (ROCK) inhibitor Y-27632 for corneal endothelial dysfunction. The clinical five-year follow-up data showed that the corneal transparency, corneal thickness, and visual acuity of 10 cases in 11 patients recovered well [14, 15]. However, transplanted CECs still depend on healthy donor corneas, and cultured CECs may eventually transform into a fibroblastic morphology or lose the ability to proliferate [16]. Diverse alternative corneal endothelial-like seed cell sources have been developed, including corneal stromal stem cells [17], human umbilical cord blood mesenchymal stem cells [18], fetal bone-marrow-derived endothelial progenitor cells [19], skin-derived precursors [20], and pluripotent stem cells (PSCs) [21]. Recently, we reported the novel strategy of injecting human embryonic stem cell (hESC)-derived corneal endothelial precursors and verified the long-term efficiency in a non-human primate animal model [22]. However, the differentiation protocols are varied, with unclear mechanisms [23, 24]. Moreover, the purity of the derived CECs cannot be definitively measured due to the lack of specific corneal endothelial markers, leading ultimately to unstable therapeutic effect [25, 26]. Furthermore, all these procedures are still far from clinical use, given the regulatory approval involved in clinical use and the clinically validated functionality associated with maintaining corneal transparency.

Ectopic cell transplantation is a promising pathway for cell therapy. For example, the oral mucosal epithelium is widely used as an equivalent substitute for the corneal epithelium in the clinical treatment of limbal stem cell deficiency, which provides strong evidence for the feasibility of ectopic cell transplantation. Previously, similar attempts have been made to treat corneal endothelial dysfunction, such as the use of vascular endothelial cells [27–29]. However, in the monkey model of bullous keratopathy, vascular endothelial cell transplantation could only partially reverse corneal thickness and transparency and was accompanied by immunological rejection. Consequently, the outcomes of vascular endothelial cell transplantation were still unsatisfactory. The promising ectopic alternative cells should better simulate the physiological characteristics of the corneal endothelium and replace its tissue function.

Here, we describe a strategy of ectopic cell transplantation for the treatment of corneal endothelial dysfunction using the retinal pigment epithelium (RPE) as an alternative seed cell source. hESC/human induced pluripotent stem cell (hiPSC)-derived RPE cells have been used for the treatment of retinal degenerative diseases, and several clinical trials have reported the long-term safety, efficacy, and tolerability of hESC/hiPSC-derived RPE cell transplantation [30–36]. Therefore, we cultured rabbit CECs and RPE cells ex vivo, representing hexagonal morphology with expressions of ZO1 and ATP1A1. When transplanted, both the primary rabbit RPE cells and hESC-derived RPE cells recovered corneal transparency and corneal thickness similar to those of CECs although there were still some issues with RPE cell transplantation for the treatment of corneal endothelial dysfunction, such as pigmentation and the risk of immune rejection and neovascularization. This study is the first to report the effectiveness of RPE cells for the treatment of corneal endothelial dysfunction, suggesting the feasibility of hESC-derived RPE cells as an equivalent substitute for CECs.

## Methods

### Animals

Non-pigmented and pigmented male rabbits were purchased from Jinan Xilingjiao Breeding Center (Shangdong, China) and housed and cared for in the animal facility of Shandong Eye Institute in accordance with the Principles of Laboratory Animal Care. Non-pigmented rabbits (3.5 kg, 6 months old) were used to establish the model of corneal endothelial dysfunction, while three-month-old non-pigmented and pigmented rabbits were used for primary rabbit RPE cells and CECs culture. All animal experiments followed the Association for Research in Vision and Ophthalmology Statement for the Use of Animals in Ophthalmic and Vision Research, which is in accordance with the guidelines and regulations approved by the Ethics Committee of the Shandong Eye Institute (W202202220025).

### Primary culture of rabbit RPE cells and CECs

Rabbits were euthanatized, and the eyeballs were stored at 4℃ for the subsequent culture of primary rabbit RPE cells and CECs. As previously described [37, 38], primary RPE cells were isolated and cultured for subsequent study. Briefly, the eyes were immersed in saline containing 400 U/mL gentamicin sulfate (CISEN Pharmaceutical Co., Shandong, China) for at least 30 min. Next, the intact corneas were separated and stored in Dulbecco’s modified Eagle’s medium (DMEM) (Corning, Manassas, VA, USA) for subsequent primary CEC culture. Excess tissues, including the lens, vitreous, and retina, were removed to obtain the optic cup, in which RPE cells were still attached on the inner side. Primary RPE cells were detached by 0.25% trypsin–EDTA (Sigma-Aldrich, Missouri, USA) for 1 h at 37 ℃, then gently isolated from Bruch’s membrane and triturated into a cell suspension. RPE cells were cultured in a medium consisting DMEM, 10% fetal bovine serum (FBS) (Gibco, Grand Island, NY, USA), 1% penicillin/streptomycin (PS, Corning), 10 μM Y-27632 (Sigma-Aldrich), 10 μM nicotinamide (NAM, Sigma-Aldrich), and 1 μM SB431542 (Millipore, Boston, Massachusetts, USA) at 37 ℃ under 5% CO_2_. A total of about 1.6 × 10^4^ RPE cells could be obtained from one single eye of rabbits, and 8 × 10^3^ cells were seeded in one well of a 24-well plate. Cultured RPE cells were observed and checked microscopically every other day. Contaminant cells (such as fibroblasts) were removed mechanically. The cultures used for experimentation were confluent and exhibited a typical cobblestone-like morphology.

Primary rabbit CECs were cultured according to a previously reported method [39]. Briefly, Descemet’s membrane (DM) and corneal endothelium were separated under a microscope and cultured in DMEM supplemented with 10 μM Y-27632 overnight. Next, CECs were digested from DM with 0.6 U/mL of collagenase I (Sigma-Aldrich) for 1 h and cultured in a medium including DMEM, 10% FBS, 1% PS, 2 ng/mL human basic fibroblast growth factor (bFGF, R&D System, Minneapolis, USA), 1% insulin-transferrin-selenium (ITS, Gibco), 1 μM SB431542, and 10 μM Y-27632 at 37℃ under 5% CO_2_. Cells from two intact corneas were seeded in one well of a 12-well plate.

The morphologies of the primary RPE cells and CECs were observed using an inverted contrast phase microscope (Nikon TE 2000‐U, Nikon, Tokyo, Japan). Cells were subcultured when they reached confluence and cultivated primary cells at passage 2 were used for the following transplantation.

### hESC culture and differentiation

The human embryonic stem cell line H1 was maintained in the serum-free mTeSR1 medium (StemCell Technologies, Vancouver, Canada) in plates coated with growth factor-reduced Matrigel (Corning) at 37 °C under 5% CO_2_. As previously described [40], RPE cells were derived from the hESCs. Briefly, hESCs (2 × 10^4^ cells) were seeded on 1% Matrigel-coated dishes and cultured in mTeSR1 for 10 days. Then, cells were grown in a differentiated medium consisting of Dulbecco's modified Eagle medium/Nutrient Mixture F-12 (DMEM/F12, Invitrogen, Carlsbad, California, USA), 15% (vol/vol) KnockOut serum (Invitrogen), 2 mM glutamine (Invitrogen), 1 × nonessential amino acids (Invitrogen), 0.1 mM β-mercaptoethanol (Sigma-Aldrich), and 1 × antibiotic–antimycotic (Invitrogen). Moreover, 10 mM NAM and 50 nM chetomin (CTM, Sigma-Aldrich) were added to the differentiated medium for the first 10–15 days of differentiation. After retention in DM only for more than three weeks, differentiated RPE cells were obtained. The morphologies of the hESCs and hESC-derived RPE cells were observed using a Nikon TE 2000‐U.

### Intracameral cell transplantation and postoperative examinations

The rabbit corneal endothelial dysfunction model was performed as previously described [22, 41]. In short, rabbits were anesthetized with intramuscular ketamine hydrochloride (40 mg/kg, Gutian Pharmaceutical Co., Fujian, China) and intravenous pelltobarbitalum natricum (50 mg/kg, Sinopharm Chemical Reagent Co., Shanghai, China). The central corneal endothelium in the right eye of each rabbit was mechanically stripped from the DM using a 20-gauge soft silicone needle (Inami, Tokyo, Japan). Depending on the types of transplanted cells at the next stage, a curettage model of different sizes was adopted, a diameter of 7 mm was applied to primary RPE cells and CECs transplantation, and a diameter of 9 mm for hESC-derived RPE cells. Next, after irrigating the cell debris with saline, the surgeon injected heparin sodium (625 U/mL, Qianhong Bio-pharma Co., Changzhou, China) into the anterior chamber to reduce inflammation. Finally, the incision was sutured, and the model was established. The right eyes of corneal endothelial-damaged rabbits (n = 3) without cell injection were used as a negative control, and the right eyes of normal rabbits (n = 3) were used as a positive control.

Cells including primary rabbit RPE cells, CECs, and hESC-derived RPE cells were dissociated with Accutase (StemCell Technologies, Vancouver, Canada) for 15 min at 37 ℃ and then gently triturated into cell suspension. Next, 3 × 10^5^ primary RPE cells (n = 3), 3 × 10^5^ primary CECs (n = 3), and 8 × 10^5^ hESC-derived RPE cells (n = 3) in 250 μL DMEM supplemented with 100 μM Y-27632 were injected into the anterior chamber immediately after closing the incision. The rabbits were still under anesthesia after surgery and were kept in the eye-down position on the operating table for 3 h to promote cell attachment. For postoperative care, all operative eyes were treated with topical medication for 14 days. Specifically, tobramycin and dexamethasone eye drops (Novartis, Basel, Switzerland) with 10 mM Y-27632 were administered four times a day. Meanwhile, local subconjunctival injections of a 1:1 mixture of 5 mg/mL dexamethasone sodium phosphate (CISEN Pharmaceutical Co.) and 0.5 mg/mL atropine sulfate (Kingyork Co., Tianjin, China) were administered once daily.

Corneal clarity, central corneal thickness, and intraocular pressure of operative eyes on days 1, 3, 7, 14, and 30 were monitored and measured using slit-lamp microscopy (SL-D7, Topcon, Tokyo, Japan), optical coherence tomography (OCT, Fremont, USA), and a tonometer (Tono-Pen AVIA, Reichert, NY, USA), respectively.

### Quantitative real-time reverse transcription polymerase chain reaction (qRT-PCR)

The relative differential mRNA expressions of rabbit RPE cells and CECs were monitored by qRT-PCR analysis. Total RNA was extracted using the MiniBEST Universal RNA Extraction Kit (TakaRa, Tokyo, Japan). Next, complementary DNA (cDNA) was synthesized by HiScript III RT SuperMix for qPCR (Vazyme, Nanjing, China), and qRT-PCR was performed using SYBR Green qPCR Master Mix (Vazyme) according to the manufacturer’s protocol. The quantified data were analyzed and normalized to glyceraldehyde-3-phosphate dehydrogenase (GAPDH). The primers used in the qRT-PCR are listed in Table 1.

**Table 1:** Primer sequences for quantitative reverse transcription-polymerase chain reaction

Gene name	Forward	Reverse
*Rabbit TJP1*	TGACCGGAGAAGTTTCGAGAAC	ATCGGTCCAGAGCATCAGCTT
*Rabbit ATP1A1*	AGAGTGGTGTCTCATTCGACAAA	GCACAGCTCGATGCATTTC
*Rabbit MITF*	TGAAGCAAGAGCGTTGGCTAA	CCCTTGTTCCAGCGCATATC
*Rabbit RPE65*	CGAAGTGATTCAGGCCAAGTC	CGAAGTGATTCAGGCCAAGTC
*Rabbit BEST1*	TGGTGACCGTAGCCGTGTAC	TCAGCCAGCCGACATAAAAGA
*Rabbit CRELBP*	AGATCAACTTCAAGGTCGGAGAAG	CGCGGGTCCAGTAGGTCTT
*Rabbit OTX2*	GTCCAGGGTACAGGTGTGGTTT	CCACTTGCTCCACTCTCTGAACT
*Rabbit GAPDH*	CGCCTGGAGAAAGCTGCTAA	CCCCAGCATCGAAGGTAGAG
*Human CD200*	CGCGGTCTGTGAGGTCACT	CGCGGTCTGTGAGGTCACT
*Human S100A4*	CGCGGTCTGTGAGGTCACT	CGCGGTCTGTGAGGTCACT
*Human SLC4A11*	GGACATCGCACGCAGGTT	CGTCATTGAGAGACCCGAAAG
*Human AQP1*	AACCCTGCTCGGTCCTTTG	CGCGGTCTGTGAGGTCACT
*Human TCF8*	TCCATGCTTAAGAGCGCTAGCT	GTATCTTGTCTTTCATCCTGATTTCCA
*Human COL8A2*	CCGGCCACCTATACCTACGAT	TCCTGAAAAGGAGGAGTGGATGTA
*Human OTX2*	CCGGGAGAGGACGACGTT	CCGGGAGAGGACGACGTT
*Human BEST1*	CCGGGAGAGGACGACGTT	GAAACTGCCGCCCAACTAGA
*Human MITF*	TCCGAAAGTTGCAACGAGAA	CCGTGGATGGAATAAGGGAAA
*Human ETS1*	CATGGACTGTGGTCATGAGTCCT	CATGGACTGTGGTCATGAGTCCT
*Human HO1*	ACACCCAGGCAGAGAATGCT	CGAAGACTGGGCTCTCCTTGT
*Human PRDX1*	GTGTGCCCCACGGAGATC	CATGGGTCCCAGTCCTCCTT
*Human PRDX6*	TGCCTGGAGCAAGGATATCAA	GTCACAGGCATGCCCTTTTC
*Human GAPDH*	CATGTTCGTCATGGGTGTGAA	CATGGACTGTGGTCATGAGTCCT

### Immunofluorescent staining

Cultured primary rabbit RPE cells, CECs, and hESC-derived RPE cells were fixed with 4% paraformaldehyde (PFA) (Biosharp, Anhui, China) for 10 min. After incubating in 0.3% Triton X-100 (Beyotime Biotechnology, Shanghai, China) for 5–15 min, all species of cells were incubated with 5% bovine serum albumin (BSA) (Boster Biological Technology, Wuhan, China) for 1 h to block nonspecific binding sites at room temperature then treated with primary antibodies (Table 2) at 4 ℃ overnight. After incubating with Alexa Fluor 488- or 594-conjugated secondary antibodies (Invitrogen, Carlsbad, California, USA) (Table 2) for 1 h at room temperature, nuclei were stained with 4,6-diamidino-2-phenylindole (DAPI) (Beyotime Biotechnology), and fluorescence images were then captured using the Echo Revolve-100-G (ECHO, San Diego, California, USA).

**Table 2:** Antibodies for immunofluorescence staining

Antibody	Supplier	Code	Dilution
Rabbit polyclonal to ZO1	Thermo Fisher	40-2200	1:100
Rabbit monoclonal to ATP1A1	Abcam	ab76020	1:150
Mouse monoclonal to MITF	Abcam	ab3201	1:100
Rabbit polyclonal to RPE65	Abcam	ab235950	1:200
Mouse monoclonal to α-SMA	Abcam	ab7817	1:100
Mouse monoclonal to human nuclei	Abcam	ab191181	1:150
Mouse monoclonal to ZO1	Thermo Fisher	33-9100	1:50
Mouse monoclonal to ATP1A1	Abcam	ab7671	1:100
Mouse monoclonal to RPE65	Thermo Fisher	MA1-16578	1:100
Mouse monoclonal to Vimentin	Abcam	ab8978	1:100
Donkey anti-mouse IgG-AF488 (H + L)	Thermo Fisher	A-21202	1:300
Donkey anti-mouse IgG-AF594 (H + L)	Thermo Fisher	SA5-10168	1:300
Donkey anti-rabbit IgG-AF488 (H + L)	Thermo Fisher	A-21206	1:300
Donkey anti-rabbit IgG-AF594 (H + L)	Thermo Fisher	SA5-10040	1:300

Normal rabbit eyeballs were obtained post-euthanasia for in situ immunofluorescent staining of the RPE and corneal endothelium. Briefly, the cornea was removed from the eyeball and fixed with 4% PFA for 12 min then stored at 4 ℃ until further use. The eye cup was obtained after removing the segments of the eye, including the iris, lens, and vitreous body. After incubating with 4% PFA for 30 min, the neurosensory retina and sclera were removed while the RPE-Buch’s membrane-choriocapillaris complex (RBCC) was acquired and stored at 4 ℃ as previously described [42]. Subsequently, in situ rabbit cornea and RBCC were permeabilized with 0.5% Triton X-100 for 5–15 min. After washing rabbit tissues with PBS three times, they were blocked in 2.5% BSA for 1 h at room temperature and incubated with primary antibodies (Table 2) overnight at 4 ℃. Both the rabbit cornea and RBCC were washed to remove excess primary antibodies the next day and then incubated with secondary antibodies (Table 2) for 1 h at room temperature. The nuclei were stained with DAPI, and the fluorescence images were observed using a laser scanning confocal microscopy (LSM 800, Zeiss, Jena, Germany).

For the immunofluorescent staining of transplanted hESC-derived RPE cells, primary rabbit RPE cells, and CECs, the corneas with the transplanted cells mentioned above were obtained after euthanasia and fixed with 4% PFA for 12 min. The following staining procedures were consistent with those of rabbit corneal endothelium in situ.

### Na+/K+-ATPase activity

Primary RPE cells and CECs were cultured and collected at passage 2 to determine their Na+/K+-ATPase activities. Their Na+/K+-ATPase activities were tested using the indicated Na+/K+-ATPase activity kits (Solarbio, Beijing, China) according to the manufacturer’s protocol.

### Cell permeability

Primary rabbit RPE cells and CECs at passage 1 were implanted in the apical side of the filter (Transwell, Corning) and their cell permeabilities were monitored using the horseradish peroxidase (HRP) tracing technique when the cells reached confluence for 5 days. HRP (50 μg/mL) in 200 μL culture medium was added to the apical side of the filter, while basal compartment medium was replaced with 500 μL culture medium without tracer. Cells were incubated at 37 ℃, and then basal compartment medium was collected at 5 min, 10 min, 15 min, and 30 min. Next, 195 μL o-phenylenediamine (Sigma-Aldrich) was reacted with the collected culture medium with tracer for 1 min, and 25 μL H_2_SO_4_ were immediately added to terminate the reaction. The color in each well changed from blue to yellow. Tracers were quantified by OD at 492 nm using a microtiter plate reader, and permeability was calculated as Flux = (X)B/(Y)i/A where (X)B represented counts in the basal chamber (μg), (Y)i was the initial concentration in the apical chamber (μg/mL), and A was the area of the filter (cm^2^).

### Electron microscopy

The RBCC and rabbit corneal endothelium were examined by scanning electron microscopy and transmission electron microscopy, respectively. The RBCC and rabbit corneal endothelium were immersed and preserved in fixative (Servicebio, Wuhan, China) at 4 ℃ and postfixed with 1% OsO_4_ in 0.1 M PB (pH 7.4) for 2 h at room temperature, avoiding light. After rinsing in 0.1 M PB (pH 7.4) three times, the tissues were dehydrated in a graded ethanol (Sinopharm Group Chemical Reagent Co. Ltd, China) series. For scanning electron microscopy preparation, the tissues were dried with a critical point dryer (Quorum, UK). Specimens were attached to metallic stubs using carbon stickers and sputter-coated with gold for 30 s before examination with scanning electron microscopy (Hitachi, Japan). For transmission electron microscopy preparation, the tissues were embedded in resin (EMBed 812). Ultrathin sections (60–80 nm thin) were fished out onto cuprum grids with formvar film and were stained with 2% uranium acetate saturated alcohol solution for 8 min as well as 2.6% lead citrate for 8 min. After drying overnight at room temperature, the cuprum grids were observed under transmission electron microscopy (Hitachi), and images were taken.

### Enzyme linked immunosorbent assay (ELISA)

The anterior chamber humors of the hESC-RPE cell transplanted rabbits and non-injected corneal endothelial-damaged rabbits were collected on day 30 after surgery, and the concentrations of vascular endothelial growth factor (VEGF) and pigment epithelium-derived factor (PEDF, Kete, Jiangsu, China) were quantified using the indicated ELISA kits according to the manufacturer’s instructions.

### Statistical analysis

All data obtained from at least three independent experiments were expressed as the mean ± standard error of the mean for the values. A student’s t-test or one-way ANOVA was used to compare the mean values between the groups using SPSS (v.19.0, Chicago, IL, USA), while *P* < 0.05 and *P* < 0.01 were considered as the significance thresholds.

## Results

### Comparison of non-pigmented rabbit RPE cells and CECs in situ

**Fig. 1:**
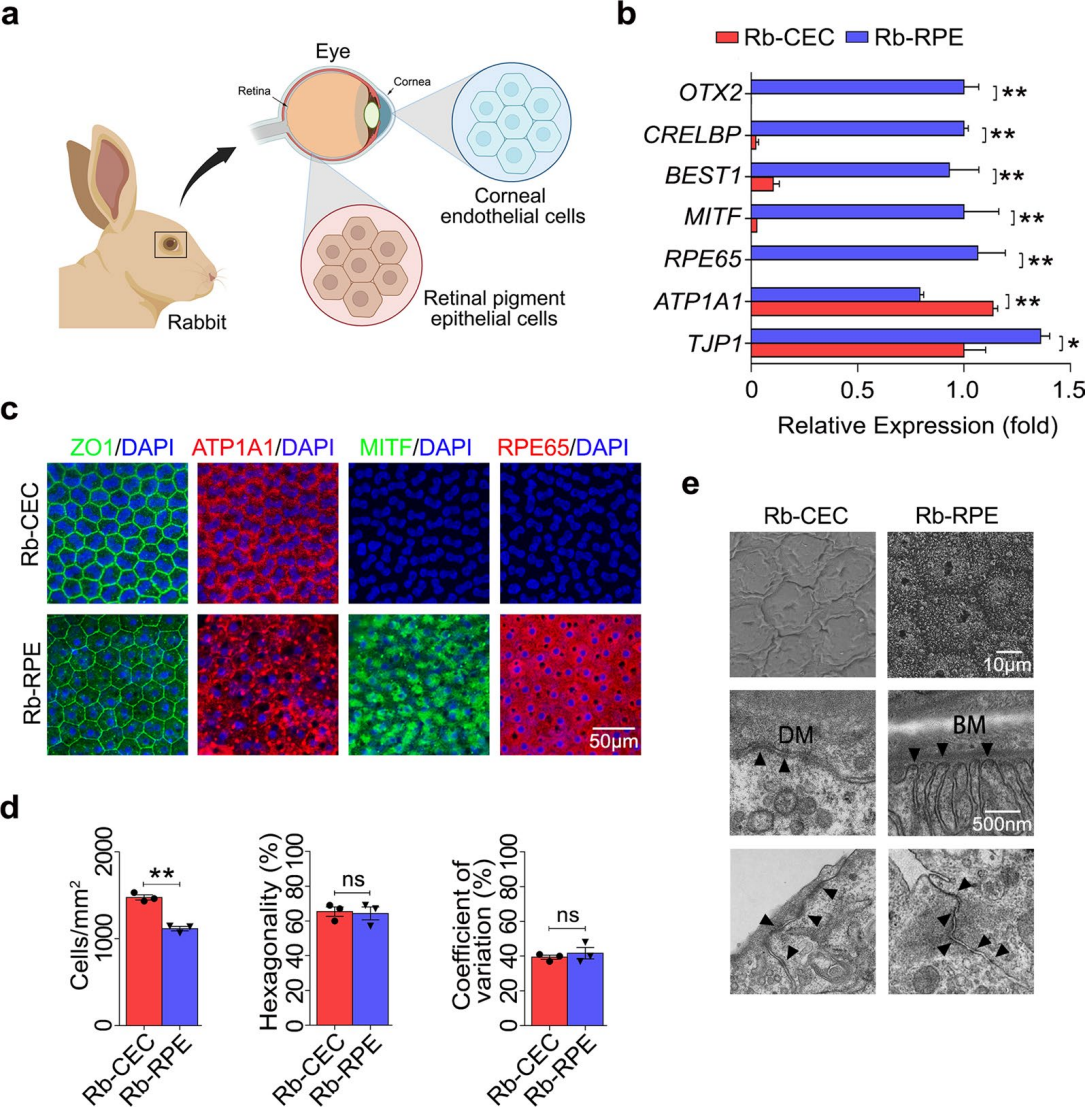
Similarities and differences between non-pigmented rabbit RPE cells and CECs in situ. **a** Schematic diagram of the morphology and location of RPE cells and CECs in rabbits created with BioRender.com. **b** qRT-PCR analysis of OTX2, CRELBP, BEST1, MITF, RPE65, ATP1A1, and TJP1 between Rb-RPE and Rb-CEC. Quantification represented the levels of relative mRNA expressions normalized to GAPDH. **c** Representative immunofluorescent images of Rb-RPE and Rb-CEC in situ were collected, including ZO1 (green), ATP1A1 (red) and retinal pigment epithelial markers MITF (green), and RPE65 (red). Nuclei were stained with DAPI (blue) (scale bar: 50 μm). **d** Cell density, hexagonality, and coefficient of variation analysis were based on ZO1 immunostaining. **e** Scanning electron microscope showed a regular hexagonal shape in both Rb-RPE and Rb-CEC, which were well formed and with distinct cell boundaries (scale bar: 10 μm) (Upper). Transmission electron microscope showed that both types of cells were attached to their respective basement membrane, named BM and DM, by hemidesmosomal junctions (▲) (scale bar: 500 nm) (Middle). Adjacent cells were joined with numerous well-developed tight junctions (▲) (scale bar: 500 nm) (Lower). Data are mean ± SEM. All results were obtained from three independent experiments. Significance (*P < 0.05, **P < 0.01 and ns: nonsignificant) relative to Rb-CEC. RPE, retinal pigment epithelium; CEC, corneal endothelial cell; qRT-PCR, quantitative real-time reverse transcription polymerase chain reaction; GAPDH, glyceraldehyde-3-phosphate dehydrogenase; BM, Bruch’s membrane; DM, Descemet’s membrane; Rb-CEC, rabbit CECs; Rb-RPE, rabbit RPE cells; SEM, standard error of the mean

Both the corneal endothelium and RPE are monolayers of hexagonal cells involved in maintaining normal visual function (Fig. 1a). To evaluate the similarities and differences of RPE cells and CECs, we isolated and studied the in situ RPE and corneal endothelium. The analysis of qRT-PCR revealed that RPE cells expressed TJP1, ATP1A1, and RPE-related genes, including RPE65, MITF, BESTROPHIN, CRELBP, and OTX2, at mRNA levels. Compared with CECs, RPE cells exhibited higher TJP1 (*P* < 0.05) and lower ATP1A1 (*P* < 0.01) (Fig. 1b). Dual immunofluorescent staining showed that both RPE cells and CECs displayed tightly hexagonal intercellular borders and monolayer cellular morphologies of regular size and shape, accompanied by positive staining of ATP1A1 (Fig. 1c). Not surprisingly, RPE-related markers of RPE65 and MITF were positively expressed in RPE cells, compared to CECs (Fig. 1c). IgG isotype controls were imaged to demonstrate the specificity of the signal (Additional file 1: Fig. S1a). The statistical analysis, based on images of in situ ZO1 immunostaining, showed that there were no significant differences in hexagonality (*P* > 0.05) and coefficient of variation (*P* > 0.05), while the cell density of RPE cells was clearly less than that of CECs (*P* < 0.01) (Fig. 1d).

To further verify the similarities and differences between RPE cells and CECs, we performed ultrastructural analyses of the RBCC and the CEC-DM complex, respectively. Scanning electron microscopy examinations revealed a similar monolayer, with continuous layers in situ formed by the two types of hexagonal cells. Both RPE cells and CECs appeared healthy and in good shape, with well-defined cell boundaries and tightly opposed cell junctions. In addition, the apical surface of RPE cells was covered with microvilli (Fig. 1e). Transmission electron microscopy showed that CECs adhere to DM with hemidesmosome attachments, much as RPE cells attach to Bruch’s membrane. Moreover, both CECs and RPE cells were tightly attached to neighboring cells by numerous desmosomes (Fig. 1e).

### Cultivation of primary non-pigmented rabbit RPE cells and CECs

**Fig. 2:**
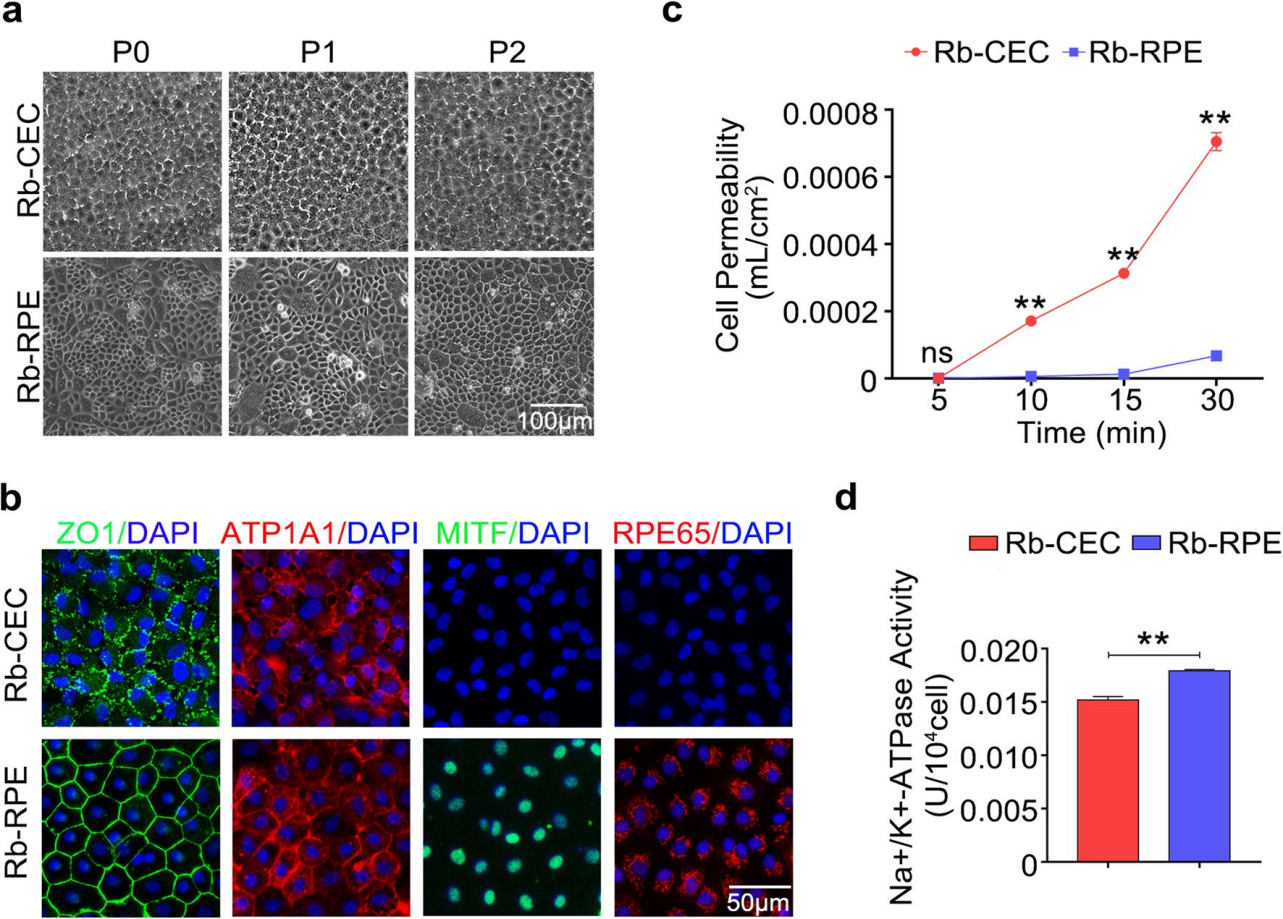
Confluent cultures of primary RPE cells and primary CECs taken from non-pigmented rabbit eyeball. **a** Cell morphology of Rb-CEC and Rb-RPE at passages 0–2 were assessed with an inverted phase-contrast microscope (scale bar: 100 μm). **b** Representative immunofluorescence staining images of corneal endothelial markers ZO1 (green), ATP1A1 (red), retinal pigment epithelial markers MITF (green), and RPE65 (red) in Rb-RPE and Rb-CEC. Nuclei were stained with DAPI (blue) (scale bar: 50 μm). **c** Cell permeabilities of Rb-RPE and Rb-CEC using HRP tracer at 5 min, 10 min, 15 min, and 30 min, respectively. **d** The Na + /K + -ATPase activities of Rb-RPE and Rb-CEC at passage 2. Data are mean ± SEM. All results were obtained from three independent experiments. Significance (**P < 0.01, ns: nonsignificant) relative to Rb-CEC. RPE, retinal pigment epithelium; CEC, corneal endothelial cell; Rb-CEC: rabbit CECs; Rb-RPE, rabbit RPE cells; SEM, standard error of the mean

Primary non-pigmented rabbit RPE cells amplified, organized, and formed a tightly connected monolayer hexagonal structure during ex vivo culture at passages 0–2, consistent with primary CECs (Fig. 2a). Furthermore, immunofluorescent staining showed positive expressions of ZO1 and ATP1A1 in primary RPE cells of passage 2, as well as in CECs, while RPE cells were confirmed with positive staining of RPE65 and MITF (Fig. 2b and Additional file 1: Fig. S1b). Since RPE cells have shown morphological similarities to CECs, we tested the barrier functions and pumping functions of these two kinds of primary cells. As shown in Fig. 2c, the value of cell permeability in RPE cells was obviously weaker than that in CECs at 10 min, 15 min, and 30 min (*P* < 0.01), while there was no significant difference at 5 min (*P* > 0.05). Moreover, Na + /K + -ATPase activity of RPE cells was higher than that of CECs (*P* < 0.01) (Fig. 2d).

### Intracameral injection of cultured non-pigmented RPE cells and CECs

**Fig. 3:**
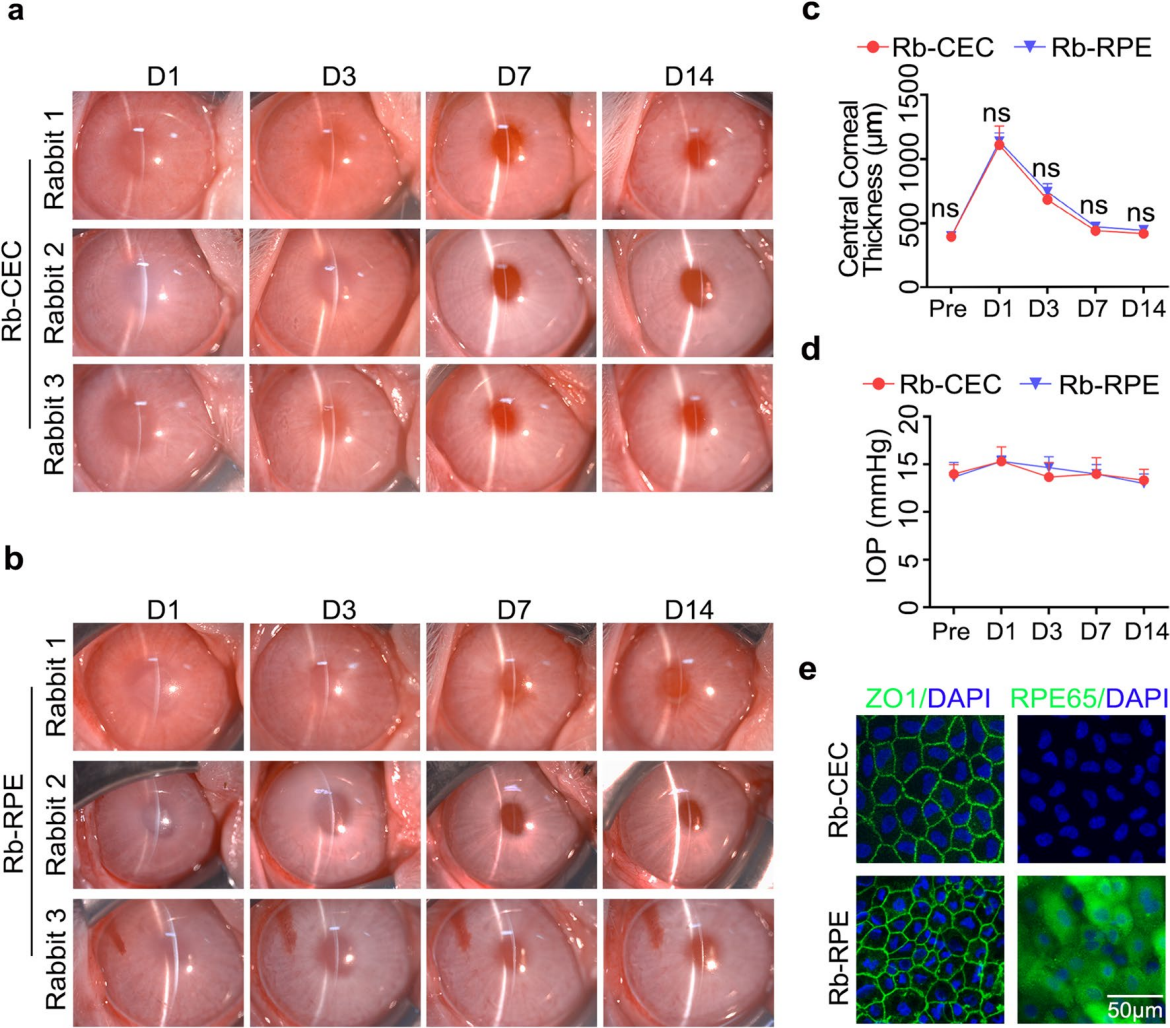
Comparisons of transplantation of primary non-pigmented rabbit RPE cells and CECs in restoring corneal endothelial dysfunction. **a**, **b** Corneal transparency of rabbits injected with non-pigmented Rb-CEC and Rb-RPE were observed by a slit lamp microscope at days 1, 3, 7, and 14, respectively. **c** OCT image-based central corneal thickness analysis. **d** IOP was measured by tonometer at days 1, 3, 7, and 14, respectively. **e** Immunofluorescence staining of ZO1 (green) and RPE65 (green) in the central area of corneas transplanted with non-pigmented Rb-RPE and Rb-CEC at day 14 postoperatively. Nuclei were stained with DAPI (blue) (scale bar: 50 μm). Data are mean ± SEM. In vivo experiments were performed using three independent animals per group. Significance (ns: nonsignificant) relative to Rb-CEC. RPE, retinal pigment epithelium; CEC, corneal endothelial cell; IOP, intraocular pressure; OCT, optical coherence tomography; Rb-CEC, rabbit CECs; Rb-RPE, rabbit RPE cells; SEM, standard error of the mean

To evaluate the therapeutic effects of primary non-pigmented rabbit RPE cells on corneal endothelial dysfunction, a quantity of 3 × 10^5^ cells supplemented with 100 μM Y-27632 were injected into the anterior chamber of central corneal-scraped rabbit, with primary CECs as the control. Rabbits injected with RPE cells exhibited gradual corneal transparency after surgery, consistent with those injected with CECs (Fig. 3a and b). Consistently, no significant difference in corneal central thickness was discovered between rabbits transplanted with non-pigmented RPE cells and CECs within 14 days after surgery (*P* > 0.05) (Fig. 3c). However, non-injected endothelial-damaged rabbits showed persistent corneal edema within 14 days after mechanical injury (Additional file 2: Fig. S2a). Moreover, intraocular pressure was not increased by intracameral injection of either RPE cells or CECs (Fig. 3d).

To further determine the roles played by primary non-pigmented RPE cells, immunofluorescent staining with functional marker ZO1 and RPE-related marker RPE65 was performed on corneas with transplanted cells at day 14 postoperatively. Compared to CECs, transplanted RPE cells formed a similar regular distribution of ZO1, with positive staining of RPE65 (Fig. 3e and Additional file 1: Fig. S1c).

### Therapeutic effects of intracameral injection of primary pigmented RPE cells

**Fig. 4:**
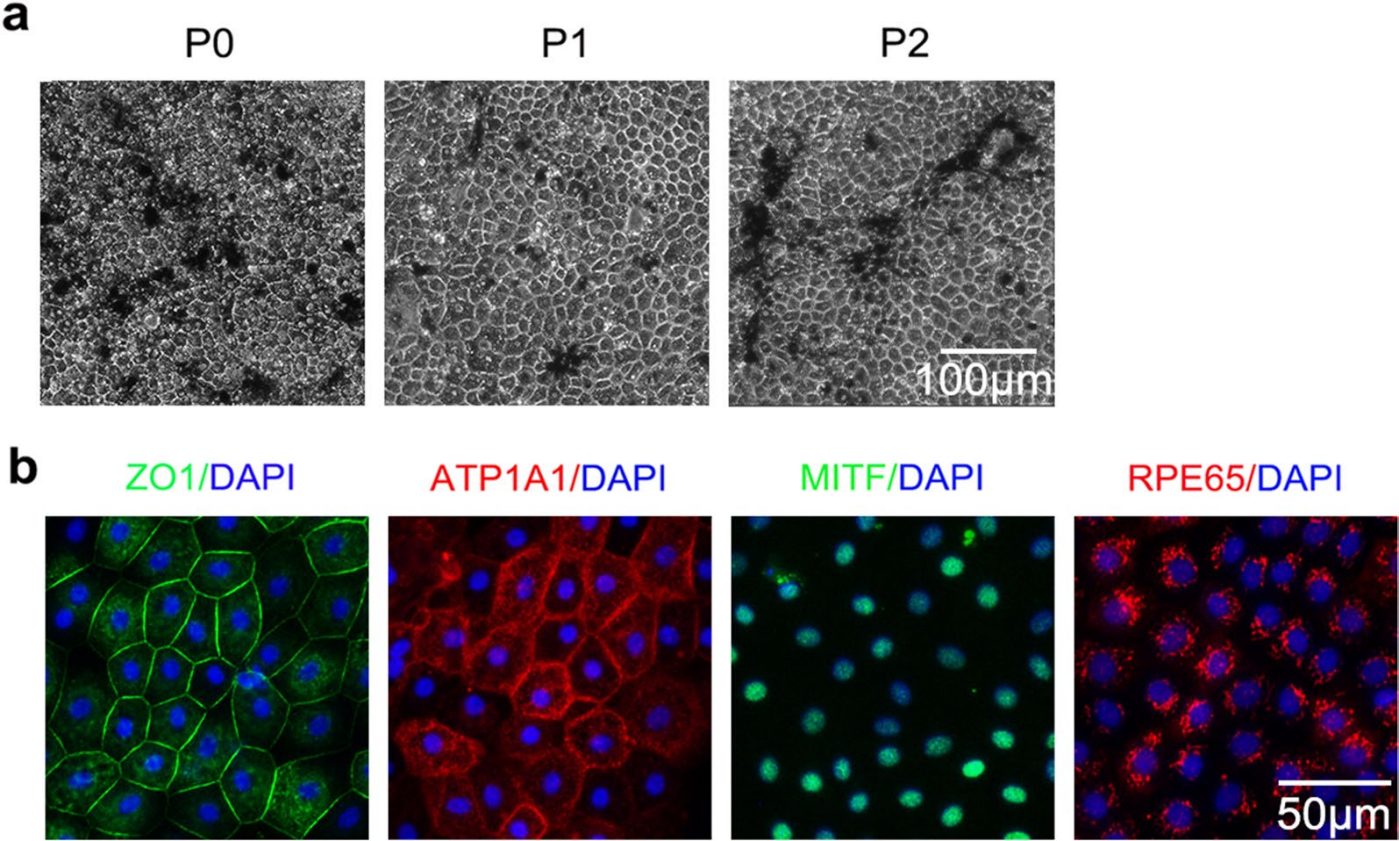
Characterization of primary pigmented RPE cells ex vivo. **a** Cell morphology of primary pigmented RPE cells at passages 0–2 (scale bar: 100 μm). **b** Representative images of functional markers ZO1 (green), ATP1A1 (red) and RPE-related markers MITF (green), and RPE65 (red) in pigmented RPE cells cultured ex vivo were recorded by immunofluorescence staining. Nuclei were stained with DAPI (blue) (scale bar: 50 μm). All results were obtained from three independent experiments. RPE, retinal pigment epithelium

**Fig. 5:**
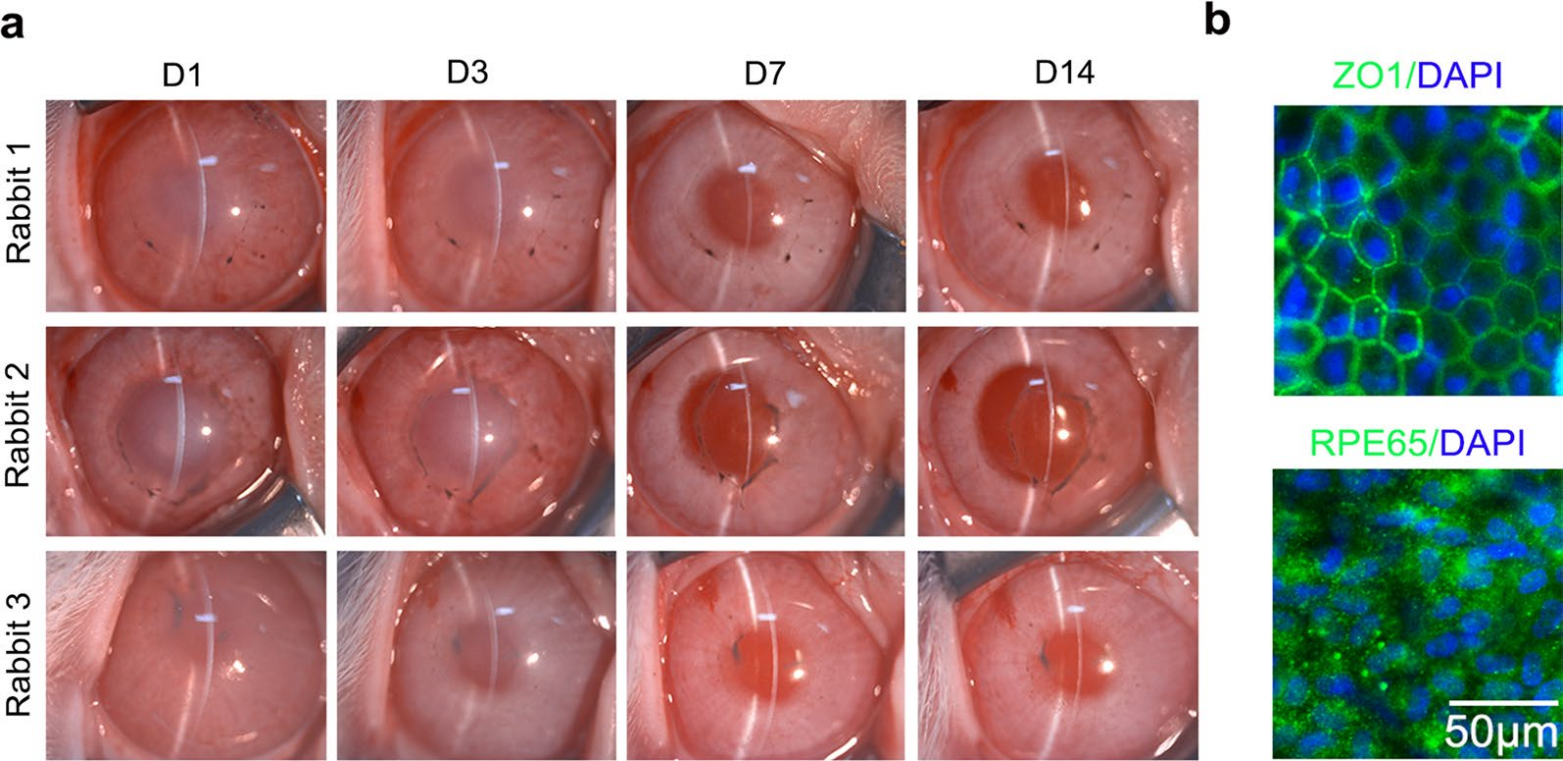
Therapeutic effects of primary pigmented RPE cells for corneal recovery. **a** Corneal transparency was observed at days 1, 3, 7, and 14 postoperatively. **b** Transplanted pigmented RPE cells were stained with ZO1 (green) and RPE65 (green) at day 14 after surgery. Nuclei were stained with DAPI (blue) (scale bar: 50 μm). In vivo experiments were performed using three independent animals per group. RPE, retinal pigment epithelium

Human RPE cells contain melanin and appear dark brown. To verify the therapeutic effects of pigmented RPE cells for corneal endothelial dysfunction, primary pigmented RPE cells were obtained and cultured, showing similar characteristics under the same culture conditions as non-pigmented RPE cells, except for substantial pigmentation (Fig. 4a and b). Primary pigmented RPE cells were injected into the anterior chamber of the central corneal-scraped rabbits to estimate the effects for corneal endothelial dysfunction. The corneas with transplanted cells achieved a rapid improvement of corneal clarity and remained stable from day 7 after operation, accompanied by pigmentation on the transplanted area, exhibiting extensively distributed functional marker ZO1 and RPE-related marker RPE65 at day 14 postoperatively (Fig. 5a and b). IgG isotype controls are shown in Additional file 1: Fig. S1d, e.

### Intracameral injection of hESC-derived RPE cells

**Fig. 6:**
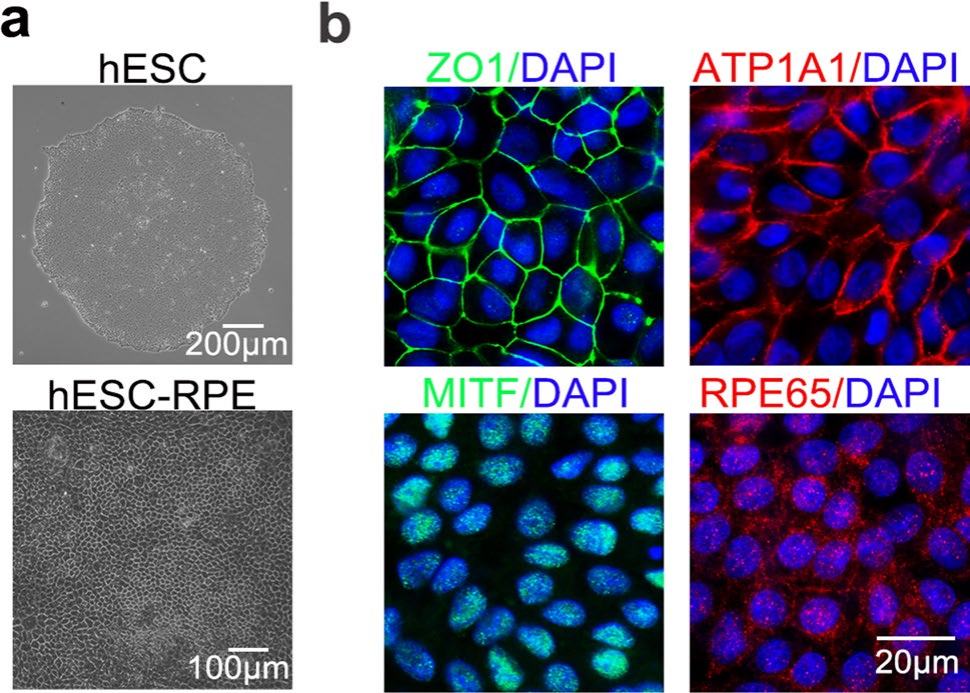
Characterization of hESC-derived RPE cells in vitro. **a** Cell morphology of hESCs (scale bar: 200 μm) and hESC-derived RPE cells (scale bar: 100 μm), respectively. **b** Representative immunostaining images of hESC-derived RPE cells (scale bar: 20 μm). All results were obtained from three independent experiments. hESC, human embryonic stem cell; RPE, retinal pigment epithelium

**Fig. 7:**
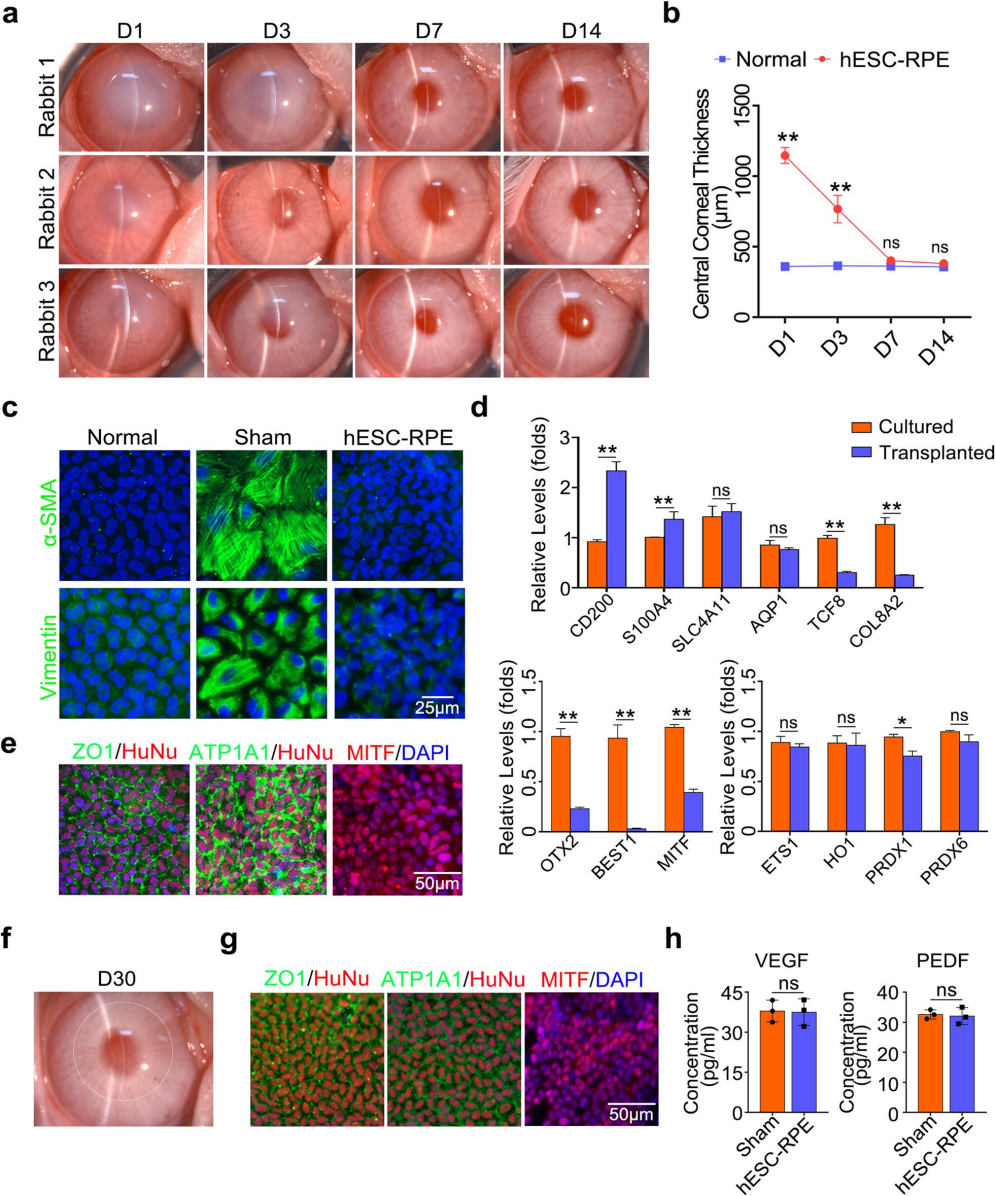
Therapeutic effects of hESC-derived RPE cells for the treatment of corneal endothelial dysfunction. **a** Corneal transparency of rabbits was measured by a slit lamp microscope at days 1, 3, 7, and 14 after cell injection. **b** OCT image-based central corneal thickness analysis. **c** Immunofluorescence staining images of α-SMA (green) and Vimentin (green) in the transplanted area 7 days after surgery. Nuclei were stained with DAPI (blue) (scale bar: 25 μm). **d** qRT-PCR analysis of CEC related genes, RPE related genes, and antioxidant genes between cultured hESC-derived RPE cells in vitro and transplanted hESC-derived RPE cells. Quantification represented the levels of relative mRNA expressions normalized to GAPDH. **e** ZO1 (green), ATP1A1 (green) and MITF (red) were stained in the central area of the cornea 14 days after surgery. The transplanted cells were stained with human specific antibody human nuclei (HuNu) (red). Nuclei were stained with DAPI (blue) (scale bar: 50 μm). **f** Slit-lamp photograph of a rabbit cornea injected with hESC-derived RPE cells at day 30 postoperatively. The white dotted circle indicates the range of transplanted cells with slight pigmentation. **g** Immunofluorescence staining images of ZO1 (green), ATP1A1 (green), and MITF (red) in the transplanted area 30 days after surgery. The transplanted cells were stained with human specific antibody human nuclei (HuNu) (red). Nuclei were stained with DAPI (blue) (scale bar: 50 μm). **h** Concentrations of VEGF and PEDF at day 30 after surgery. Data are mean ± SEM. In vivo experiments were performed using three independent animals per group. Significance (*P < 0.05, **P < 0.01, ns: nonsignificant) relative to control. hESC, human embryonic stem cell; RPE, retinal pigment epithelium; OCT, optical coherence tomography; hESC-RPE, hESC-derived RPE cells; qRT-PCR, quantitative real-time reverse transcription polymerase chain reaction; GAPDH, glyceraldehyde-3-phosphate dehydrogenase; Sham, non-injected corneal endothelial damaged group. VEGF, vascular endothelial growth factor; PEDF, pigment epithelium-derived factor

To further verify the availability of human RPE cells, we explored whether hESC-derived RPE cells could alleviate corneal endothelial dysfunction. As shown in Fig. 6a and b, hESCs were induced into the polygonal RPE cells, which had positive staining of RPE-related markers MITF and RPE65 and exhibited regular and continuous distributions of ZO1 and ATP1A1 at the cell membrane. Images of IgG isotype controls are shown in Additional file 1: Figure S1f. Then, 8 × 10^5^ hESC-derived RPE cells were intracamerally injected into the rabbit model. The transplanted hESC-derived RPE cells rapidly improved corneal clarity and restored corneal thickness within seven days (*P* > 0.05) (Fig. 7a and b). Endothelial-mesenchymal transition (EnMT) is one of the major challenges of cell therapy for treating corneal endothelial damage. We demonstrated that transplanted cells in the central damaged area were negative for the classical EnMT marker α-SMA and weakly positive for Vimentin seven days after operation, which were obviously expressed in the non-injected endothelial-damaged group (Fig. 7c). Next, immunostaining results also showed that transplanted cells were positive for human specific antibody human nuclei (HuNu), RPE-related marker MITF, and corneal endothelial functional markers ZO1 and ATP1A1 at day 14 postoperatively (Fig. 7e and Additional file 1: Fig. S1g). qRT-PCR analysis showed that relative expression of CEC markers such as CD200, S100A4 clearly increased in transplanted hESC-derived RPE cells at day 14 after surgery (*P* < 0.01), accompanied by significant decreased expressions of specific RPE markers OTX2, BEST1, and MITF. However, some CEC-related functional genes such as SLC4A11 and AQP1 showed no significant changes, while the relative expression of TCF8 and COL8A2 decreased significantly postoperatively (*P* < 0.01). Moreover, there were no significant changes in the expression levels of antioxidant-related genes, including ETS1, HO1, and PRDX6 (*P* > 0.05) (Fig. 7d).

To verity the long-term effects, rabbits transplanted with hESC-derived RPE cells were followed up to one month, showing normal corneal thickness and slight pigmentation in the central cornea (Fig. 7f). Immunofluorescent staining showed the transplanted cells were positive for HuNu, ZO1, ATP1A1 and MITF (Fig. 7g). Moreover, we detected the concentrations of VEGF and PEDF in the aqueous humor of rabbits with transplanted hESC-derived RPE cells, and the concentrations did not increase significantly compared with that of non-injected corneal damaged rabbits (*P* > 0.05) (Fig. 7h).

## Discussion

Cell-based therapeutics represent a promising strategy to relieve the global shortage of corneal donor tissues. In past decades, the outcomes of diverse seed cell sources for corneal endothelial dysfunction have been verified, but clinical translation still needs more consideration. In the present study, we demonstrated for the first time that RPE cells are capable of being an equivalent substitute of corneal endothelium for the treatment of corneal endothelial dysfunction. Intracameral injection of RPE cells, including primary rabbit RPE cells and hESC-derived RPE cells, achieved stable therapeutic recovery of corneal thickness and transparency in the rabbit model of corneal endothelial dysfunction that was similar to CEC injection.

There is uncertainty about CEC regenerative therapy so far [43], although several preclinical animal experiments have demonstrated the effectiveness of hESC/hiPSC-derived CECs in improving corneal edema [21, 22, 44]. Firstly, the mechanism and the signaling pathway in the protocol from hESCs/hiPSCs into CECs are unclear, with co-cultured technology and a variety of small molecule chemical compound used in the process [23, 24]. Moreover, the identification of CECs is difficult due to a shortage of specific corneal endothelial markers, which will result in poor purity of differentiated cells [25, 26]. Briefly, ZO1, ATP1A1, CD200 and S100A4 are always used for identification of CECs [45–49], but they are also expressed by many other tissues. Therefore, cell purification is still the key problem affecting clinical transformation. RPE cells play a critical role in retinal development and metabolism, choroidal formation and maintenance, and retinal homeostasis [50, 51]. RPE cell defects will lead to irreversible retinal degenerative diseases such as age-related macular degeneration, Stargardt’s macular dystrophy, and more serious retinal degeneration with choroidal atrophy [52, 53]. Cell or tissue transplantation is considered one of the most promising methods for visual restoration, particularly in advanced-stage disease [30]. Various different sources of cells for RPE cell therapy have been attempted as potential seed cells, including hESC-derived RPE cells [31–33], hiPSC-derived RPE cells [34, 35] and allogenic RPE cells [54]. Notably, the scheme of hESCs/hiPSCs differentiation into RPE cells is more mature, efficient, and repeatable, and differentiated RPE cells with high purity and good function are easier to obtain [35]. Moreover, several clinical trials have reported the long-term safety, efficacy, and tolerability of hESC/hiPSC-derived RPE cell transplantation [31, 36], and many more are underway [30]. Thus, among the studies of cell-based therapeutics for ophthalmic diseases, RPE cell transplantation is one of the most mature and leading strategies, particularly the transplantation of hESC/hiPSC-derived RPE cells.

CECs regulate the corneal hydration state by functioning both as a leaky barrier and active ionic pump [55–57], bearing several similarities to RPE cells. Specifically, RPE cells are polarized into apical and basolateral plasma membrane domains separated by tight junctions, which, among other things, enable RPE cells to form the outer blood-retinal barrier between the retina and the systemic circulation [58, 59]. Functionally, RPE cells can mediate bidirectional transport between the retina and the choroid, appearing to selectively transport biomolecules such as nutrients, ions, and water, which protect the health and integrity of the outer retina [59, 60]. In this study, we detected morphological comparability and functional differences between RPE cells and CECs. Structurally, both types of cells in situ showed a regular hexagonal shape that was well formed with tightly opposed cell junctions, and adhered to the basement membrane through hemidesmosomes, in which RPE cells were slightly larger, with a lower cell density. Subsequently, we demonstrated that RPE cells had a stronger barrier and greater ionic pumping capacity than CECs functionally. Moreover, it should be noted that CECs and RPE cells had different embryological origins, with CECs originating from the neural crest cells [61] and RPE cells originating from neuroectoderm [62]. Even so, our findings led us to consider the interesting hypothesis that RPE cells might act as an alternative to CECs in the treatment of corneal endothelial dysfunction.

We also assessed the efforts of RPE cell therapy using the rabbit corneal endothelial dysfunction model in line with previous reports [22, 41]. Both primary rabbit RPE cells and hESC-derived RPE cells showed obvious advantages in the treatment of corneal endothelial dysfunction, which showed no significant difference compared to primary rabbit CECs. Subsequently, to avoid the confounding factor of autogenous rabbit corneal endothelial cell proliferation, the non-injected damaged endothelial rabbits were observed at various time-points, showing persistent corneal edema and obvious EnMT compared to cell transplanted rabbits. qRT-PCR analysis showed shifts in the gene expression patterns of transplanted hESC-derived RPE cells, hinting at the possibility that the RPE cells underwent functional adaptive changes under the microenvironment of the anterior chamber. However, it is worth mentioning that hESC-derived RPE cells still expressed the typical MITF one month after transplantation, suggesting that the cell fate of RPE cells was not changed after transplantation, with other changes needing further experimental verification. In addition, we confirmed that the transplanted hESC-derived RPE cells survived and maintained normal corneal transparency for one month, similar to primary cells, with no significant side effects, suggesting the human RPE cells could replace CECs to maintain stroma hydration.

There are several issues with RPE cell transplantation for the treatment of corneal endothelial dysfunction. First, we verified that both the primary rabbit pigmented RPE cells and hESC-derived RPE cells could resolve corneal edema and recover corneal thickness. However, normal human RPE cells contain melanin and appear dark brown, playing the roles of protection and visual generation [63]. Meanwhile, hyperpigmentation would certainly impair corneal clarity and visual function. These suggest that pigmentation-knockout RPE cells could be used to completely restore corneal visual function. Second, the in situ RPE cells are known to secrete many cytokines, including PEDF and VEGF [64, 65], which possibly increase the risks of immune rejection and neovascularization after RPE cell transplantation for corneal endothelial dysfunction. Indeed, we detected the concentration of VEGF and PEDF in the aqueous humor of rabbits with transplanted hESC-derived RPE cells at day 30 after surgery, and the concentration did not increase significantly compared with that of non-injected corneal damaged rabbits. In addition, we determined that there was no obvious immune rejection or neovascularization in the rabbits with the intracameral injection of RPE cells within one month. Similarly, the transplantation of vascular endothelial cells increased the level of VEGF in the aqueous humor, but, this did not promote the obvious neovascularization in the iris and cornea [66].

Our study is an effective attempt to treat corneal endothelial dysfunction by RPE cell transplantation. However, it should be mentioned that there are still some limitations. First, unlike human CECs, rabbit CECs can proliferate to repair corneal endothelial damage. Comparatively, a non-human primate animal model can better simulate human corneal endothelial dysfunction, where CECs have shown limited regenerative capacity. Further experiments in non-human primates are needed to verify the long-term effectiveness and safety, the effects of the pigmentation-knockout RPE cells, and the fate change of the transplanted hESC-derived RPE cells. Moreover, the previous studies had reported multiple differentiation protocols for RPE cells derived from different hESC/hiPSC lines [40, 67–69]. It is necessary to verify the therapeutic effects of RPE cells derived from different cell sources and protocols in future studies.

## Conclusion

Our study describes a novel therapeutic method for corneal endothelial dysfunction using RPE cells as an equivalent substitute for CECs. We successfully performed RPE cell injection into the rabbit model of corneal endothelial dysfunction and confirmed that RPE cells were capable of a rapid and stable restoration of rabbit corneal clarity. We determined that hESC-derived RPE cell transplantation offered a feasible approach for the treatment of corneal endothelial dysfunction.

## Supplementary Information

**Additional file 1: Figure S1.** Immunofluorescent stainings of IgG isotype controls. **a** Non-pigmented rabbit CECs and RPE cells in situ were stained with mouse IgG isotype controls (scale bar: 50 μm). **b** Cultured primary non-pigmented rabbit CECs and RPE cells were stained with mouse IgG isotype controls (scale bar: 50 μm). **c** Transplanted primary non-pigmented rabbit CECs and RPE cells were stained with mouse IgG isotype controls (scale bar: 50 μm). **d** Cultured primary pigmented RPE cells were stained with mouse IgG isotype controls (scale bar: 50 μm). **e** Transplanted primary pigmented RPE cells were stained with mouse IgG isotype controls (scale bar: 50 μm). **f** Mouse IgG isotype controls and rabbit IgG isotype controls in cultured hESC-derived RPE cells (scale bar: 20 μm). **g** Mouse IgG isotype controls and rabbit IgG isotype controls in transplanted hESC-derived RPE cells (scale bar: 50 μm). Nuclei were stained with DAPI (blue). CEC, corneal endothelial cell; RPE, retinal pigment epithelium; Rb-CEC, rabbit CECs; Rb-RPE, rabbit RPE cells.

**Additional file 2: Figure S2.** Corneal transparency of mechanical damaged rabbits and normal rabbits. **a** Corneal transparency of mechanical damaged rabbits without cell injection (negative control) was measured by a slit lamp microscope at days 1, 3, 7, and 14 after surgery. **b** Corneal transparency of normal rabbits (positive control) was measured by a slit lamp microscope at days 1, 3, 7, and 14. In vivo experiments were performed using three independent animals per group.

## Data Availability

The data that support the findings of this study are available from the corresponding author upon reasonable request.

## Abbreviations

RPE: Retinal pigment epithelium
CECs: Corneal endothelial cells
PSCs: Pluripotent stem cells
hESCs: Human embryonic stem cells
hiPSCs: Human induced pluripotent stem cells
DMEM: Dulbecco’s modified Eagle’s medium
FBS: Fetal bovine serum
PS: Penicillin/streptomycin
NAM: Nicotinamide
DM: Descemet’s membrane
bFGF: Basic fibroblast growth factor
ITS: Insulin-transferrin-selenium
DMEM/F12: Dulbecco’s modified Eagle medium/Nutrient Mixture F-12
CTM: Chetomin
OCT: Optical coherence tomography
cDNA: Complementary DNA
GAPDH: Glyceraldehyde-3-phosphate dehydrogenase
qRT-PCR: Quantitative real-time reverse transcription polymerase chain reaction
PFA: Paraformaldehyde
BSA: Bovine serum albumin
DAPI: 4,6-Diamidino-2-phenylindole
RBCC: RPE-Buch’s membrane-choriocapillaris complex
HRP: Horseradish peroxidase
HuNu: Human specific antibody human nuclei
AMD: Age-related macular degeneration
PEDF: Pigment epithelium-derived factor
VEGF: Vascular endothelial growth factor
BM: Bruch’s membrane
ELISA: Enzyme linked immunosorbent assay
