# Supplementary figures and images for: Ex vivo cultivated retinal pigment epithelial cell transplantation for the treatment of rabbit corneal endothelial dysfunction
Source: Eye Vis (Lond). 2023 Aug 2;10:34. doi: 10.1186/s40662-023-00351-4 (PMC10394777; doi:10.1186/s40662-023-00351-4)

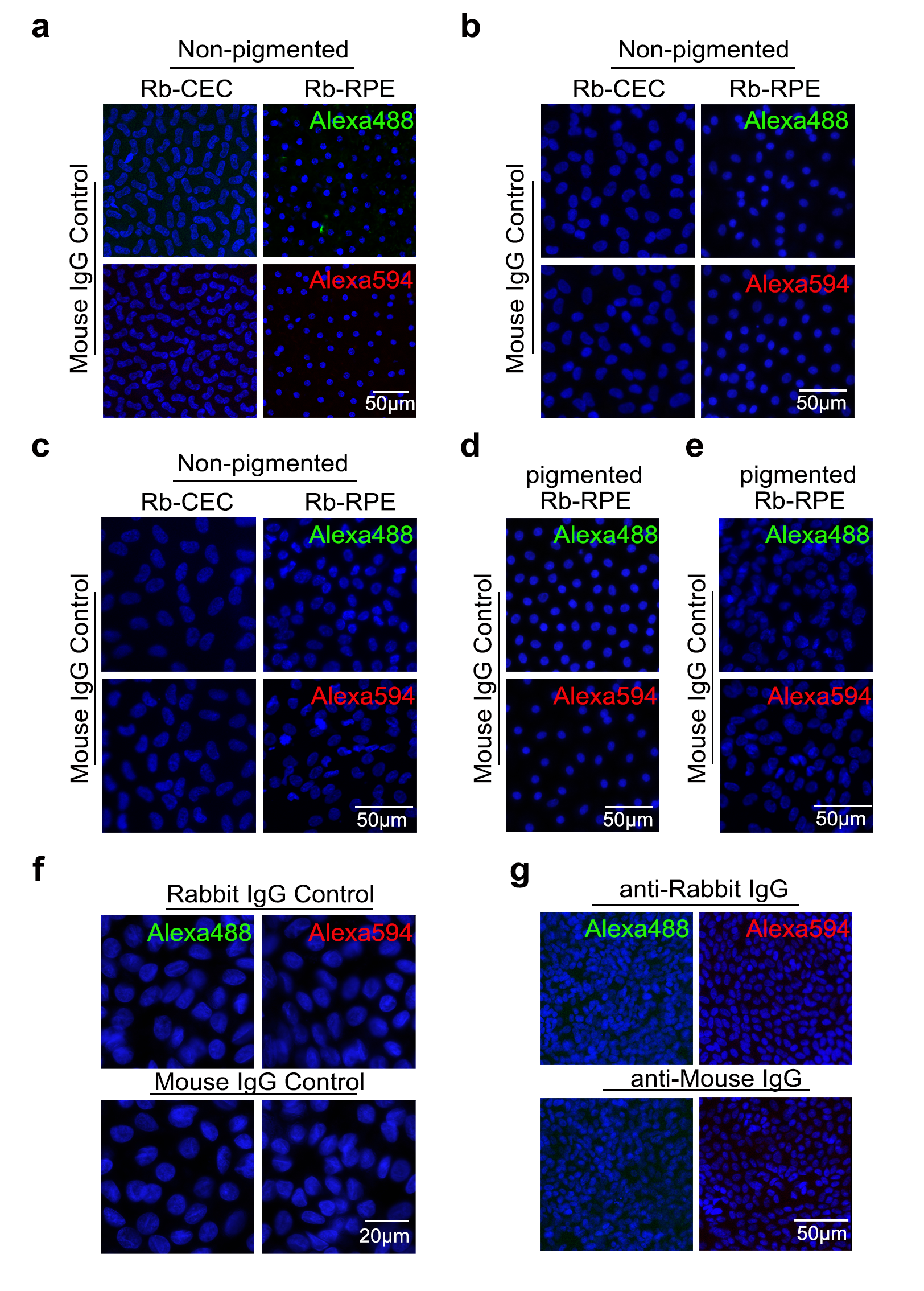

Supplement: Supplementary file 1 — Additional file 1: Figure S1. Immunofluorescent stainings of IgG isotype controls. a Non-pigmented rabbit CECs and RPE cells in situ were stained with mouse IgG isotype controls (scale bar: 50 μm). b Cultured primary non-pigmented rabbit CECs and RPE cells were stained with mouse IgG isotype controls (scale bar: 50 μm). c Transplanted primary non-pigmented rabbit CECs and RPE cells were stained with mouse IgG isotype controls (scale bar: 50 μm). d Cultured primary pigmented RPE cells were stained with mouse IgG isotype controls (scale bar: 50 μm). e Transplanted primary pigmented RPE cells were stained with mouse IgG isotype controls (scale bar: 50 μm). f Mouse IgG isotype controls and rabbit IgG isotype controls in cultured hESC-derived RPE cells (scale bar: 20 μm). g Mouse IgG isotype controls and rabbit IgG isotype controls in transplanted hESC-derived RPE cells (scale bar: 50 μm). Nuclei were stained with DAPI (blue). CEC, corneal endothelial cell; RPE, retinal pigment epithelium; Rb-CEC, rabbit CECs; Rb-RPE, rabbit RPE cells. [file 40662_2023_351_MOESM1_ESM.tif]

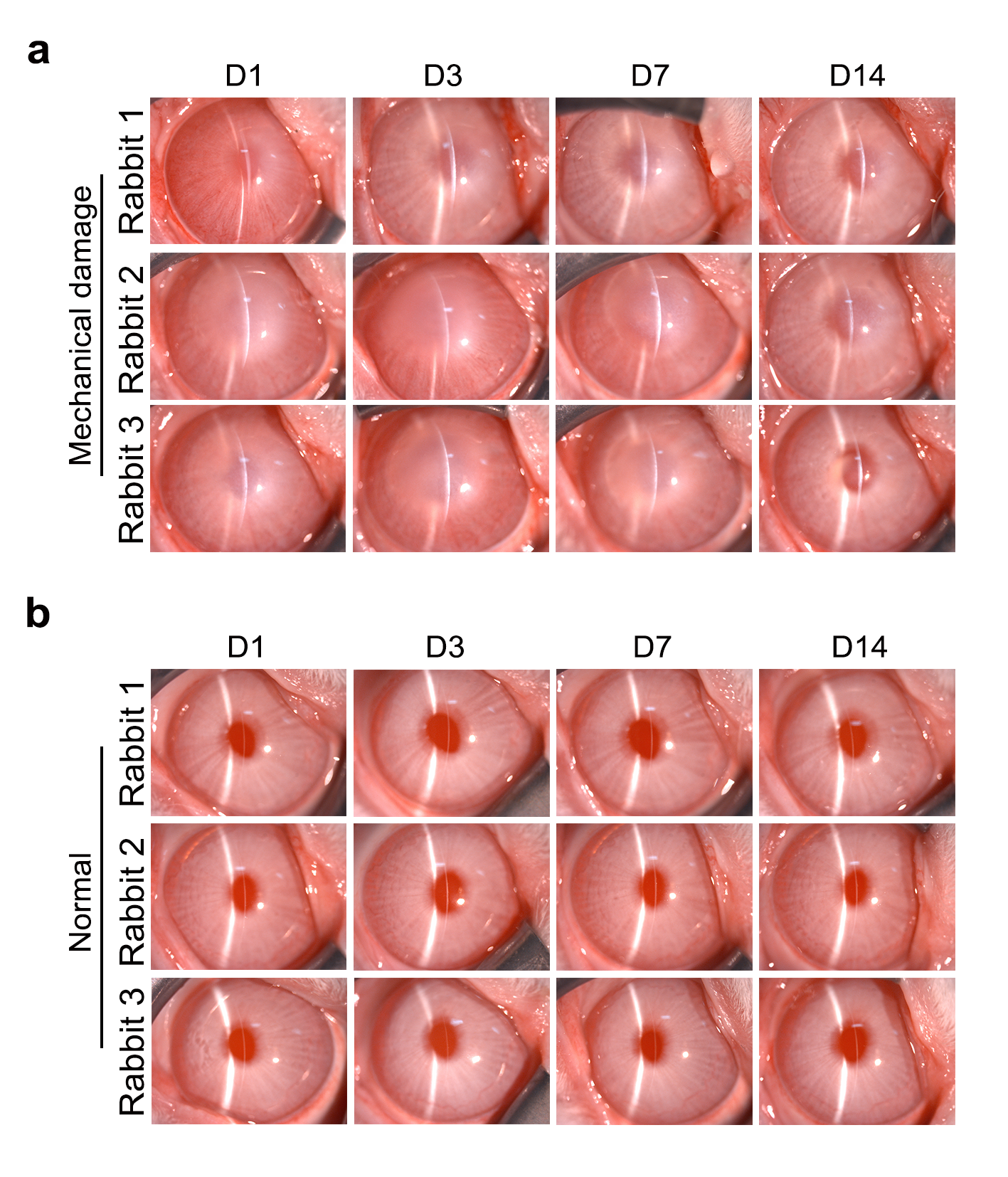

Supplement: Supplementary file 2 — Additional file 2: Figure S2. Corneal transparency of mechanical damaged rabbits and normal rabbits. a Corneal transparency of mechanical damaged rabbits without cell injection (negative control) was measured by a slit lamp microscope at days 1, 3, 7, and 14 after surgery. b Corneal transparency of normal rabbits (positive control) was measured by a slit lamp microscope at days 1, 3, 7, and 14. In vivo experiments were performed using three independent animals per group. [file 40662_2023_351_MOESM2_ESM.tif]
